# *APOE*/*TOMM* *40* genetic loci, white matter hyperintensities, and cerebral microbleeds

**DOI:** 10.1111/ijs.12615

**Published:** 2015-08-26

**Authors:** Donald M. Lyall, Susana Muñoz Maniega, Sarah E. Harris, Mark E. Bastin, Catherine Murray, Michael W. Lutz, Ann M. Saunders, Allen D. Roses, Maria del C. Valdés Hernández, Natalie A. Royle, John M. Starr, David J. Porteous, Ian J. Deary, Joanna M. Wardlaw

**Affiliations:** ^1^ Centre for Cognitive Ageing and Cognitive EpidemiologyUniversity of EdinburghEdinburghUK; ^2^ Brain Research Imaging Centre, Division of Neuroimaging SciencesUniversity of EdinburghEdinburghUK; ^3^ Department of PsychologyUniversity of EdinburghEdinburghUK; ^4^ Medical Genetics SectionUniversity of Edinburgh Centre for Genomics and Experimental Medicine and MRC Institute of Genetics and Molecular MedicineWestern General HospitalEdinburghUK; ^5^ Scottish Imaging NetworkA Platform for Scientific Excellence (SINAPSE) CollaborationDepartment of Neuroimaging SciencesThe University of EdinburghEdinburghUK; ^6^ Joseph & Kathleen Bryan Alzheimer's Disease Research CenterDepartment of NeurologyDuke University Medical CenterDurhamNCUSA; ^7^ Zinfandel Pharmaceuticals, Inc.DurhamNCUSA; ^8^ Alzheimer Scotland Dementia Research CentreEdinburghUK

**Keywords:** brain microbleeds, epidemiology, MRI, neurology, risk factors, vascular events

## Abstract

**Background:**

Two markers of cerebral small vessel disease are white matter hyperintensities and cerebral microbleeds, which commonly occur in people with Alzheimer's disease.

**Aim and/or hypothesis:**

To test for independent associations between two Alzheimer's disease‐susceptibility gene loci – *APOE* ε and the *TOMM* *40* ‘523’ poly‐T repeat – and white matter hyperintensities/cerebral microbleed burden in community‐dwelling older adults.

**Methods:**

Participants in the Lothian Birth Cohort 1936 underwent genotyping for *APOE* ε and *TOMM* *40* 523, and detailed structural brain magnetic resonance imaging at a mean age of 72·70 years (standard deviation = 0·7; range = 71–74).

**Results:**

No significant effects of *APOE* ε or *TOMM* *40* 523 genotypes on white matter hyperintensities or cerebral microbleed burden were found amongst 624 participants.

**Conclusions:**

Lack of association between two Alzheimer's disease susceptibility gene loci and markers of cerebral small vessel disease may reflect the relative health of this population compared with those in other studies in the literature.

## Introduction

There is evidence that the presence of cardiovascular disease pathology can increase the future risk of Alzheimer's disease (AD) and cognitive decline 1. White matter hyperintensities (WMH) and cerebral microbleeds (CMB) are generally considered to reflect cerebrovascular burden in ageing. They are manifestations and markers of cerebral small‐vessel disease and often co‐occur 2.

The mechanisms underlying significant association between cardiovascular and neurodegenerative pathology are unclear; however, there are three main hypotheses 1. Firstly, it is possible that cardiovascular diseases and AD/cognitive decline share common risk factors and are not mechanistically related 1. Secondly, it is possible that cardiovascular burden may expedite progression of AD/cognitive decline through promoting atherosclerosis and accumulations of amyloid‐beta plaques, and/or (thirdly) by increasing vulnerability to such pathology and lowering the threshold at which cognitive decline becomes apparent behaviorally, even in the absence of a mechanistic link 1, 3.

Two genetic risk factors for AD and age‐related cognitive decline are in the *APOE* and *TOMM40* gene loci 4. Two recent meta‐analyses reported no overall significant association between *APOE* ε4 and WMH in older adults (*P* > 0·05). Paternoster *et al*. 5 did not stratify analyses by whether participants were generally healthy or not (total *n* = 8546), whereas Schilling *et al*. 6 did (‘healthy’ *n* = 8405). However, these reports were not independent because some common data were included in both. In contrast, two meta‐analyses reported a significant association between *APOE* ε4 and CMB: Schilling *et al*. 6 (‘healthy’ *n* = 5387; *P* = 0·002) and Maxwell *et al*. 7 (total *n* = 7351; *P* < 0·01). These two reports also had a degree of overlapping datasets. A more recent individual study 8 reported similar results (*n* = 1965, *P* < 0·05).

In the above reports, the positive association between *APOE* ε4 and CMB burden was not completely consistent; other genetic predictor variables may exert independent effects. The *TOMM40* rs10524523 (‘523’) variable length poly‐T repeat has been significantly associated with brain phenotypes such as cognitive decline, independent of *APOE* genotype 9. The *TOMM40* 523 locus is characterized by a variable number of T residues (poly‐T repeats) that can be grouped into ‘short’ (<20; ‘S’), ‘long’ (20–29; ‘L’), and ‘very long’ (≥30; ‘VL’) 10. Roses 11 plotted histograms showing the distributions of poly‐T repeat lengths in different *APOE* genotypes: ε3/ε3, ε3/ε4, and ε4/ε4. The poly‐T repeat was strongly linked with the *APOE* ε haplotype; ε4 is linked to L, with ε3 linked to either S or VL alleles 4; investigating the effects of *TOMM40* 523 genotype on brain‐related phenotypes may explain some of the heterogeneity in the possible *APOE* ε4 and WMH/CMB association. This study therefore aims to contribute a large amount of relevant genetic *APOE*/*TOMM40* and WMH/CMB brain imaging data, from a sample of community‐dwelling older adults.

## Methods

### Sample and genotyping

The Lothian Birth Cohort 1936 (LBC1936) is a longitudinal sample of generally healthy community‐dwelling older adults 12. All participants were born in 1936, and most resided in the Edinburgh area of Scotland in older age. The sample received detailed cognitive, medical, and demographic assessments at the Wellcome Trust Clinical Research Facility (Edinburgh; http://www.wtcrf.ed.ac.uk) at age ∼73 years. Participants underwent detailed brain MRI around the same time 13 (mean interval = 65·0 days, SD = 39·5). Of the 866 LBC1936 participants that attended clinic assessment, 700 completed neuroimaging (mean age = 72·70, SD = 0·7). Details of LBC1936 recruitment and assessment, including aspects of possible selection bias and attrition, can be found in two cohort protocol papers by Deary *et al*. 12, 14.

Participants were genotyped by TaqMan assay for *APOE* ε (Applied Biosystems, Carlsbad, CA, USA) using DNA isolated from whole blood 12. *TOMM40* 523 was genotyped by the laboratory of Dr. Ornit Chiba‐Falek (Duke University) as described previously 15.

### Brain MRI

Participants underwent whole brain structural MRI, acquired using a GE Signa Horizon 1·5 T HDxt clinical scanner (General Electric, Milwaukee, WI, USA) equipped with a self‐shielding gradient set (33 mT/m maximum gradient strength) and manufacturer‐supplied eight‐channel phased‐array head coil, lasting around 70 min. In addition to standard structural T2‐, T2*‐, and FLAIR‐weighted MRI, the imaging protocol included a high‐resolution T1‐weighted volume sequence acquired in the coronal plane with field‐of‐view of 256 × 256 mm, imaging matrix 192 × 192 (zero‐filled to 256 × 256), 160 1·3‐mm thick slices giving 1 × 1 × 1·3‐mm voxel dimensions 13. The repetition, echo, and inversion times were 10, 4, and 500 ms respectively. The detailed protocol for WMH/CMB image processing, and intracranial/total brain volume measurement, is published by Wardlaw *et al*. 13. WMH volumes were calculated from binary masks generated by an in‐house‐developed and validated software tool written in MATLAB that applies a technique named Multispectral Colouring Modulation and Variance Identification: 1936 [MCMxxxVI 16 ]. Visual scoring of WMH was also performed using the Fazekas scale by experienced neuroradiologists.

Microhemorrhages (i.e. CMBs) were coded for number and distribution using a simplified version of the Brain Observer MicroBleed Scale [BOMBS 17 ], which considers microbleeds as small homogenous round foci of low signal intensity on T2*‐weighted images, of less than 10 mm in diameter. This rating scale is used to record the number of observed definite or possible microbleeds in the right/left hemispheres, delineated into bleeds <5 mm and 5–10 mm. Because of the relatively low frequency of CMB's in the LBC1936 sample, we examined the presence of ≥1 definite/possible microbleeds, strictly lobar microbleeds, strictly deep or infratentorial microbleeds. Any significant findings were reanalyzed as definite microbleeds only.

Inter‐ and intra‐rater reliability standards have been reported in previous work 13. Genotyping was performed blind to imaging (and vice versa). Imaging lesions were defined according to STRIVE recommendations 2. Of the 700 participants that completed brain MRI, 25 had one or more lacunar infarcts, and given this low frequency, we did not consider this variable further.

### Statistical analysis

Age in days and gender were included as covariates. An online calculator was used to perform tests of Hardy–Weinberg equilibrium and determine minor allele frequencies (http://www.had2know.com/academics/hardy‐weinberg‐equilibrium‐calculator‐3‐alleles.html). Volumetric WMH data were transformed with a natural logarithmic function to provide a more normal distribution. Data were analyzed with the IBM SPSS statistics program (version 17; IBM, Armonk, NY, USA).

Univariate general linear models tested the effects of separate *APOE* and *TOMM40* genotypes upon imaging variables. Specifically, the effects of *APOE* ε4 ‘risk’ allele presence vs. absence (i.e. ε2/ε4; ε3/ε4; ε4/ε4 vs. ε2/ε2; ε2/ε3; ε3/ε3) were tested. To assess the independent effects of *TOMM40* 523 genotype, we tested for effects of S allele frequency vs. pooled L/VL alleles (simply L*; S/S vs. S/L* vs. L*/L*) in participants with the ‘neutral’ *APOE* ε3/ε3 genotype 9 and then in the ε3/ε4 genotype subgroup, because *TOMM40* 523 genotype may interact with the ε4 allele 18.

## Results

Of the 866 LBC1936 participants that attended clinic assessment, 700 completed neuroimaging. Participants were excluded from analysis if they had Mini‐Mental State Exam scores below 24 or had not completed the test 19. [A cutoff of 24 was used because this is considered the lowest possible score within the range of ‘no cognitive impairment’, and is a common approach 20.]. This left 694 participants, of whom 624 and 636 had successful genotyping for *APOE* ε and *TOMM40* 523, respectively. Allele frequencies were in Hardy–Weinberg equilibrium for *APOE* (ε2 = 7·4%, ε3 = 77·0%; ε4 = 15·6%) and *TOMM40* (S = 40·9%, L = 15·3%, and VL = 43·9%; both *P* > 0·05). Allele frequencies are shown in Table S1.

There were no significant associations between *APOE* ε4 presence (vs. absence), or *TOMM40* 523 genotype (in any analyses), and WMH/CMB variables (all *P* > 0·05; Tables S2–S3).

## Discussion

Our findings align with previous meta‐analyses in observing no significant *APOE*/WMH association 5, 6. In terms of CMB, this report does not align with recent meta‐analyses that concluded significant deleterious effects of *APOE* ε4 6, 7. Of those meta‐analyses, Schilling *et al*. 6 observed a significant effect of *APOE* ε4 in general populations only – and not in pooled samples with neurological or vascular disorders; the lack of association in LBC1936 may be because of the relatively good health of the sample. Our findings do align with two individual studies of generally healthy older adults (included in the meta‐analyses) which reported no significant *APOE* ε4 present (vs. absent) effects [by Sveinbjornsdottir *et al*. 21 (*n* = 1725) and Jeerakathil *et al*. 22 (*n* = 368)]. There was no evidence of an independent effect of *TOMM40* 523 genotype here.

Previous significant associations in individual CMB reports may perhaps reflect a degree of type 1 error, particularly in smaller samples. Several studies report broader age ranges than examined here (71–74 years; SD = 0·7) 7. Any effect of age on CMB may be via processes *associated* with age; controlling for age statistically is unlikely to completely eradicate these effects 23, so wide age ranges could possibly contribute to spurious genetic associations.

The BOMBS instrument allows raters to note CMBs as either definite or possible [a cautious category to avoid misclassifications of mimics 17 ]. It would be interesting to examine if previously reported significant *APOE*‐CMB associations are affected when analyzed to incorporate possible microbleeds/mimics.

It is possible that the sample size examined here is not sufficiently powered to detect any possibly modest effects of *APOE* or *TOMM40* genotypes on WMH/CMB 5. It is possible that the LBC1936 sample is generally healthier when compared with other samples, exacerbated by a selection bias where healthier participants were more likely to attend brain MRI assessment 24. Generally, the LBC1936 sample is slightly restricted in range towards the upper end of general mental ability and socioeconomic status 14. In addition, *APOE* ε4 genotype has previously been associated with earlier mortality and cardiovascular disease 25: it is possible that a selection bias exists whereby healthier participants are more likely to attend cognitive or brain imaging assessment, and this may contribute to our finding no effect of *APOE/TOMM40* genotypes on WMH/CMB phenotypes with MRI.

Maxwell *et al*. 7 estimated with a ‘fail‐safe N calculation’ that null studies including at least 7700 participants would be required to attenuate their meta‐analysis *APOE* ε4/CMB association (reported *P* = 0·01) to non‐significance (i.e. *P* > 0·05). Further independent studies will help to define the more exact nature and strength of any *APOE*/CMB association in generally healthy populations.

## Authors' contributions

Designed the experiments: D. M. L., J. M. W., D. J. P., I. J. D. Analyzed the data/wrote the manuscript: D. M. L. Interpreted the results, critically revised content, and approved the final version: All authors.

## Supporting information


**Table S1.** Frequency statistics for *APOE/TOMM40* poly‐T repeat genotypes.

**Table S2.** *APOE/TOMM40* genotypes and white matter hyperintensities/cerebral microbleeds: association statistics.

**Table S3.** *TOMM40* genotypes and white matter hyperintensities/cerebral microbleeds in *APOE* subgroups: association statistics.
