# Supplementary material for: APOE/TOMM 40 genetic loci, white matter hyperintensities, and cerebral microbleeds
Source: Int J Stroke. 2015 Aug 26;10(8):1297–300. doi: 10.1111/ijs.12615 (PMC4950052; doi:10.1111/ijs.12615)
Supplement: Supplementary file 1 — Table S1. Frequency statistics for APOE/TOMM40 poly‐T repeat genotypes. [file IJS-10-1297-s001.docx]

**Supplementary Table 1** frequency statistics for *APOE/TOMM40* poly-T repeat genotypes.

|  | **TOMM40 genotype, N (%)** | | | | | |
| --- | --- | --- | --- | --- | --- | --- |
| *APOE* genotype | *S/S* | *S/L* | *S/VL* | *L/L* | *L/VL* | *VL/VL* |
| *ɛ2/ɛ2* | 1 (100) | - | - | - | - | - |
| *ɛ2/ɛ3* | 18 (23.1) | 1 (1.3) | 35 (44.9) |  | 1 (1.3) | 23 (29.5) |
| *ɛ2/ɛ4* | - | 7 (50) | - |  | 7 (50) | - |
| *ɛ3/ɛ3* | 77 (20.4) | 3 (0.8) | 198 (52.5) | 1 (0.3) | 2 (0.5) | 96 (25.5) |
| *ɛ3/ɛ4* | - | 83 (50.6) | 8 (4.9) | 1 (0.6) | 66 (40.2) | 6 (3.7) |
| *ɛ4/ɛ4* | - | - | - | 11 (100) | - | - |

*Note.* S = short, L = Long, VL = very-long.
