# Supplementary material for: APOE/TOMM 40 genetic loci, white matter hyperintensities, and cerebral microbleeds
Source: Int J Stroke. 2015 Aug 26;10(8):1297–300. doi: 10.1111/ijs.12615 (PMC4950052; doi:10.1111/ijs.12615)
Supplement: Supplementary file 2 — Table S2. APOE/TOMM40 genotypes and white matter hyperintensities/cerebral microbleeds: association statistics. [file IJS-10-1297-s002.docx]

**Supplementary** **Table 2** *APOE/TOMM40* genotypes and white matter hyperintensities/cerebral microbleeds: association statistics.

|  | *APOE* ε genotype: ɛ4+ vs. ɛ4- | | | | *TOMM40 523* genotype: S/S vs. S/L* vs. L*/L* | | | | |
| --- | --- | --- | --- | --- | --- | --- | --- | --- | --- |
|  | ɛ4-  Mean (SE) | ɛ4+  Mean (SE) | *P* | Partial  η^2^ | S/S  Mean (SE) | S/L*  Mean (SE) | L*/L*  Mean (SE) | *P* | Partial  η^2^ |
| Periventricular Left (Fazekas score) | 1.34 (0.03) | 1.33 (0.05) | 0.880 | <0.001 | 1.32 (0.07) | 1.34 (0.04) | 1.36 (0.04) | 0.849 | 0.001 |
| Periventricular Right (Fazekas score) | 1.33 (0.03) | 1.30 (0.05) | 0.665 | <0.001 | 1.31 (0.04) | 1.33 (0.04) | 1.32 (0.04) | 0.942 | <0.001 |
| Periventricular Overall (Fazekas score) | 1.36 (0.03) | 1.34 (0.05) | 0.774 | <0.001 | 1.35 (0.07) | 1.35 (0.04) | 1.37 (0.04) | 0.934 | <0.001 |
| Deep, Left (Fazekas score) | 1.04 (0.03) | 1.05 (0.05) | 0.823 | <0.001 | 1.01 (0.07) | 1.06 (0.04) | 1.09 (0.05) | 0.591 | 0.002 |
| Deep, Right (Fazekas score) | 1.01 (0.03) | 1.05 (0.05) | 0.496 | 0.001 | 0.99 (0.07) | 1.04 (0.04) | 1.04 (0.05) | 0.764 | 0.001 |
| Deep, Overall (Fazekas score) | 1.07 (0.03) | 1.08 (0.05) | 0.902 | <0.001 | 1.04 (0.07) | 1.10 (0.04) | 1.08 (0.05) | 0.726 | 0.001 |
| White matter hyperintensities alone in brain tissue volume (%; natural log-transformed) | 0.60 (0.02) | 0.63 (0.03) | 0.437 | 0.001 | 0.59 (0.05) | 0.63 (0.03) | 0.61 (0.03) | 0.688 | 0.001 |
| Untransformed median (interquartile range) | 0.66  (0.27-1.46) | 0.72  (0.39-1.44) | - | - | 0.60  (0.24-1.41) | 0.71  (0.33-1.06) | 0.69  (0.28-1.36) | - | - |
| White matter hyperintensities alone in intracranial volume (%; natural log-transformed) | 0.51 (0.02) | 0.53 (0.03) | 0.534 | 0.001 | 0.50 (0.04) | 0.53 (0.02) | 0.51 (0.03) | 0.747 | 0.001 |
| Untransformed median (interquartile range) | 0.51  (0.21-1.14) | 0.55  (0.29-1.15) | - | - | 0.44  (0.20-1.13) | 0.55  (0.27-1.22) | 0.83  (0.22-1.05) | - | - |
| ≥1 possible/definite microbleeds N (%) | 49 (10.89) | 23 (12.11) | 0.618 | <0.001 | 15 (15.6) | 36 (10.8) | 24 (8.8) | 0.415 | 0.003 |
| ≥1 possible/definite lobar microbleeds N (%) | 16 (3.56) | 7 (3.68) | 0.929 | <0.001 | 4 (4.2) | 12 (3.6) | 7 (3.2) | 0.928 | <0.001 |
| ≥1 possible/definite deep/infratentorial  microbleeds N (%) | 31 (6.89) | 7 (3.68) | 0.129 | 0.004 | 5 (5.2) | 18 (5.4) | 15 (6.8) | 0.693 | 0.001 |

*Note*. S = short allele; L* = pooled long and very-long alleles. SE = standard error. White matter hyperintensity volumetric data has been transformed with a natural logarithm function. Mean/SE data are estimated marginal means adjusted for the covariates of age (in days) and gender.
