# Supplementary material for: APOE/TOMM 40 genetic loci, white matter hyperintensities, and cerebral microbleeds
Source: Int J Stroke. 2015 Aug 26;10(8):1297–300. doi: 10.1111/ijs.12615 (PMC4950052; doi:10.1111/ijs.12615)
Supplement: Supplementary file 3 — Table S3. TOMM40 genotypes and white matter hyperintensities/cerebral microbleeds in APOE subgroups: association statistics. [file IJS-10-1297-s003.docx]

**Supplementary** **Table 3** *TOMM40* genotypes and white matter hyperintensities/cerebral microbleeds in *APOE* subgroups: association statistics.

|  | In *APOE* ɛ3/ɛ4 (‘risk’) only  S/S vs. S/L* vs. L*/L* | | | | in *APOE* ɛ3/ɛ3 only (‘neutral’) only  S/S vs. S/L* vs. L*/L* | | | | |
| --- | --- | --- | --- | --- | --- | --- | --- | --- | --- |
|  | S/L*  Mean (SE) | L*/L*  Mean (SE) | *P* | Partial  η^2^ | S/S  Mean (SE) | S/L*  Mean (SE) | L*/L*  Mean (SE) | *P* | Partial  η^2^ |
| Periventricular Left (Fazekas score) | 1.37 (0.06) | 1.35 (0.07) | 0.880 | <0.001 | 1.32 (0.08) | 1.33 (0.05) | 1.38 (0.07) | 0.745 | 0.002 |
| Periventricular Right (Fazekas score) | 1.36 (0.07) | 1.29 (0.07) | 0.468 | 0.003 | 1.31 (0.08) | 1.33 (0.05) | 1.35 (0.07) | 0.927 | <0.001 |
| Periventricular Overall (Fazekas score) | 1.38 (0.07) | 1.34 (0.07) | 0.701 | 0.001 | 1.36 (0.08) | 1.35 (0.05) | 1.40 (0.07) | 0.790 | 0.001 |
| Deep, Left (Fazekas score) | 1.08 (0.08) | 1.09 (0.08) | 0.930 | <0.001 | 0.95 (0.08) | 1.04 (0.05) | 1.05 (0.07) | 0.566 | 0.003 |
| Deep, Right (Fazekas score) | 1.10 (0.08) | 1.09 (0.08) | 0.923 | <0.001 | 0.98 (0.08) | 1.00 (0.05) | 1.02 (0.07) | 0.921 | <0.001 |
| Deep, Overall (Fazekas score) | 1.16 (0.08) | 1.09 (0.08) | 0.561 | 0.002 | 1.01 (0.07) | 1.06 (0.05) | 1.08 (0.06) | 0.742 | 0.002 |
| White matter hyperintensities alone in brain tissue volume (%; natural log-transformed) | 0.69 (0.05) | 0.60 (0.05) | 0.178 | 0.012 | 0.59 (0.05) | 0.60 (0.03) | 0.61 (0.05) | 0.966 | <0.001 |
| Untransformed median (interquartile range) | 0.81  (0.46-1.72) | 0.68  (0.34-1.26) | - | - | 0.60  (0.24-1.54) | 0.69  (0.29-1.47) | 0.67  (0.27-1.39) | - | - |
| White matter hyperintensities alone in intracranial volume (%; natural log-transformed) | 0.58 (0.04) | 0.50 (0.05) | 0.201 | 0.010 | 0.50 (0.05) | 0.50 (0.03) | 0.51 (0.04) | 0.978 | <0.001 |
| Untransformed median (interquartile range) | 0.63  (0.35-1.30) | 0.52  (0.25-1.00) | - | - | 0.44  (0.19-1.20) | 0.53  (0.22-1.16) | 0.53  (0.21-1.09) | - | - |
| ≥1 possible/definite microbleeds N (%) | 13 (14.4) | 6 (6.7) | 0.253 | 0.008 | 11 (15.07) | 15 (7.77) | 10 (8.26) | 0.188 | 0.009 |
| ≥1 possible/definite lobar microbleeds N (%) | 5 (5.6) | 1 (1.4) | 0.177 | 0.012 | 4 (5.48) | 4 (2.07) | 4 (4.08) | 0.350 | 0.006 |
| ≥1 possible/definite deep/infratentorial  microbleeds N (%) | 2 (2.2) | 4 (5.6) | 0.267 | 0.008 | 3 (4.11) | 12 (6.22) | 7 (7.14) | 0.668 | 0.002 |

*Note*. S = short allele; L* = pooled long and very-long alleles. SE = standard error. White matter hyperintensity volumetric data has been transformed with a natural logarithm function. Mean/SE data are estimated marginal means adjusted for the covariates of age (in days) and gender.
